# IRF1 amplifies HSV-1-triggered antiviral innate immunity in a feed-forward manner

**DOI:** 10.1016/j.cellin.2025.100255

**Published:** 2025-05-22

**Authors:** Ming Gao, Yining Qi, Junjie Zhang

**Affiliations:** a State Key Laboratory of Oral & Maxillofacial Reconstruction and Regeneration, Key Laboratory of Oral Biomedicine Ministry of Education, Hubei Key Laboratory of Stomatology, School & Hospital of Stomatology, State Key Laboratory of Virology and Biosafety, Medical Research Institute, Wuhan University, Wuhan 430071, Hubei, China; b Frontier Science Center for Immunology and Metabolism, Medical Research Institute, Wuhan University, Wuhan 430071, Hubei, China; c Hubei Key Laboratory of Tumor Biological Behavior, Hubei Province Cancer Clinical Study Center, Zhongnan Hospital of Wuhan University, Wuhan 430071, Hubei, China

**Keywords:** HSV-1, IRF1, MITA, STING, Antiviral innate immunity, IRF3

## Abstract

Herpes simplex virus 1 (HSV-1) is a prevalent human pathogen that establishes lifelong infection and causes a wide range of diseases. Antiviral innate immunity is critical for controlling HSV-1 replication; however, how host cells elicit a full spectrum of antiviral innate immune responses against HSV-1 remains poorly understood. Here, our studies indicate that Interferon regulatory factor 1 (IRF1) amplifies HSV-1-induced antiviral innate immunity in a feed-forward manner. Our data reveal that HSV-1 infection induces IRF1 expression, and MITA/STING contributes to the induction of IRF1 during HSV-1 infection. Moreover, IRF1 restricts HSV-1 replication dependent on its DNA-binding activity. Knockout of IRF1 significantly diminishes the induction of a large subset of interferon-stimulated genes (ISGs) critical for antiviral defense during HSV-1 infection. Notably, IRF1 interacts with IRF3, promoting its recruitment to the promoters of ISGs as well as type I and III interferons, thereby facilitating the activation of antiviral signaling. These findings uncover a novel amplifying role of IRF1 in HSV-1-induced antiviral immunity, which deepens our understanding of innate immune responses against viral infections.

## Highlights

• MITA/STING signaling contributes to the induction of IRF1 during HSV-1 infection.
• IRF1 restricts HSV-1 replication dependent on its DNA-binding activity.
• IRF1 forms a complex with IRF3 to promote the induction of interferon-stimulated genes.

## Graphical abstract

**Figure:**
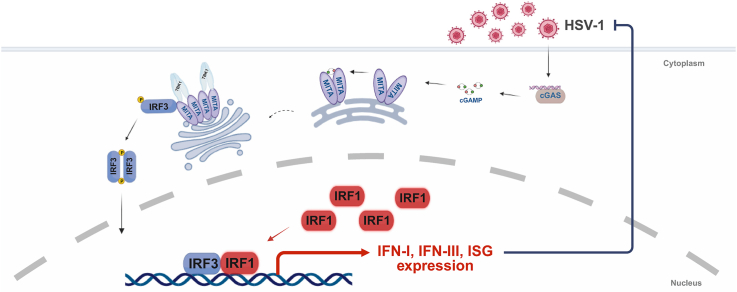

## 1 Introduction

Herpes simplex virus 1 (HSV-1) is a ubiquitous human pathogen that infects over 60% of adults globally and causes a range of diseases, from mild oral lesions to more severe manifestations such as encephalitis (Fu & Pan, 2025; Marcocci et al., 2020). After primary infection of epithelial cells, HSV-1 initiates an acute replication phase, and subsequently establishes latency in neurons of the peripheral nervous system (Marcocci et al., 2020). Under certain conditions, HSV-1 can infect the central nervous system and lead to herpes encephalitis, particularly when antiviral innate immune responses are compromised (Serrero & Paludan, 2024; Zhang & Casanova, 2024), highlighting the central role of innate immune defense in restricting HSV-1 infection.

Upon HSV-1 infection, the host innate immune system serves as the first line of defense, detecting viral components through pattern recognition receptors (PRRs) and initiating antiviral responses (Zhu & Zheng, 2020). Previous studies have revealed that HSV-1 infection engages multiple PRRs, including cGAS, RIG-I, IFI16, and TLR3, in diverse cell types, triggering antiviral innate immune responses (Chiang et al., 2018; Latif et al., 2020; Liu et al., 2016; Melchjorsen et al., 2009; Orzalli et al., 2012, 2015; Sun et al., 2013; Zhang et al., 2018; Zhao et al., 2016; Zheng et al., 2022). These immune signaling pathways converge on the activation of transcription factors such as interferon regulatory factors (IRFs) and NF-κB to drive the production of interferons (IFNs) and interferon-stimulated genes (ISGs) (Hu & Shu, 2018; Zhang & Zhong, 2022). Central to this process are IRFs, a family of transcription factors that play a crucial role in modulating antiviral responses (Wang, Zhu, et al., 2024). Among the IRFs, IRF3 and IRF7 are widely studied and have been shown to play critical roles in the induction of type I IFNs and antiviral effectors that restrict viral replication (Wang, Zhu, et al., 2024). In contrast, IRF1 is less well characterized. IRF1 consists of an N-terminal DNA binding domain (DBD), a nuclear localization signal (NLS), and a C-terminal IRF-association domain. IRF1 is activated in response to viral infections, and its DBD domain recognizes a hexanucleotide unit, “GTGAAA”, referred to as IRF-binding elements, leading to the induction of pro-inflammatory and antiviral immune responses (Feng et al., 2021). IRF1 has been shown to promote the phosphorylation of IRF3 by blocking the interaction between IRF3 and PP2A, leading to the upregulation of innate immunity, which is independent of the DNA-binding activity of IRF1 (Wang et al., 2020). However, whether IRF1, which is predominantly localized in the nucleus, potentiates antiviral innate immune responses in the nucleus dependent on its DNA-binding activity remains unexplored. Previous studies have established that IRF1 contributes to HSV-1-induced innate immune responses and restricts HSV-1 replication in vitro and in vivo (Kimura et al., 1994; Ru et al., 2014; Sharma et al., 2023; Wang et al., 2019; Xie et al., 2018). However, the precise mechanisms by which IRF1 regulates antiviral innate immunity during HSV-1 infection remain elusive.

In this study, we found that IRF1 amplifies HSV-1-induced antiviral innate immunity through a feed-forward mechanism. Our studies reveal that MITA signaling contributes to the induction of IRF1 during HSV-1 infection, and IRF1 restricts HSV-1 replication dependent on its DNA-binding activity. Notably, IRF1 interacts with IRF3, enhancing its ability to bind to the promoters of ISGs as well as type I and III interferons, thereby boosting antiviral signaling. This study uncovers a novel amplifying role of IRF1 in antiviral immunity, specifically in the context of HSV-1 infection, and provides insights into the complex regulation of innate immune responses to viral pathogens.

### 1.1 MITA contributes to the induction of IRF1 during HSV-1 infection

**Fig. 1:**
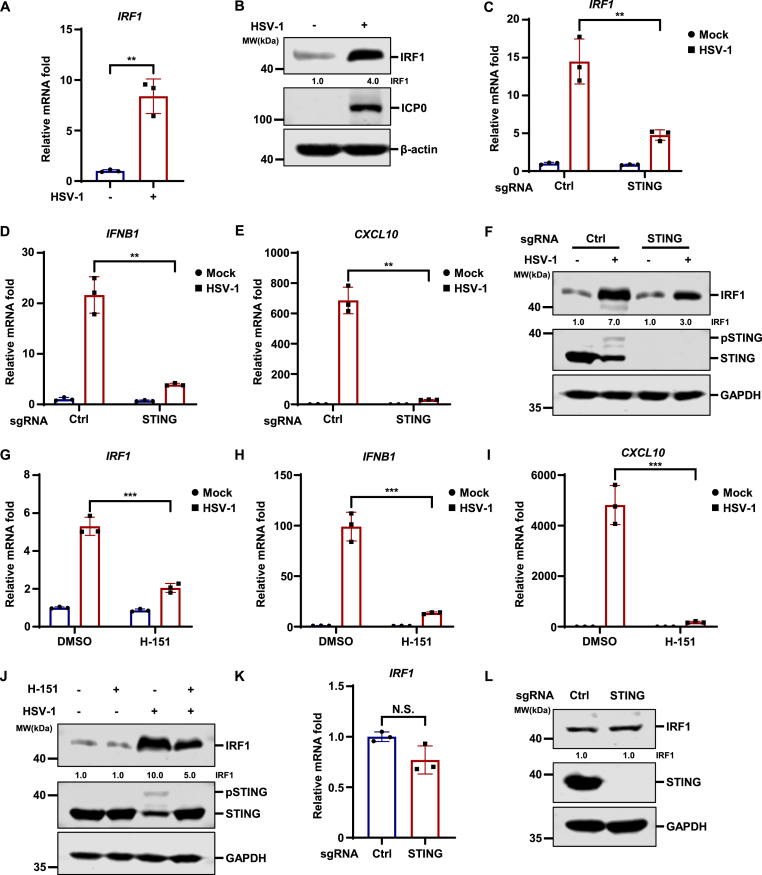
**HSV-1 infection induces IRF1 and MITA/STING contributes to IRF1 induction.** (A–B) HT1080 cells were either mock-infected or infected with HSV-1 (MOI = 5) for 8 h. The expression of *IRF1* was quantified by RT-qPCR (A), and whole cell lysates (WCLs) were collected and analyzed by immunoblotting (B). (C–E) HT1080 cells transduced with control sgRNA (Ctrl) or sgRNA targeting *MITA/STING* were mock-infected or infected with HSV-1 (MOI = 5), and the expression of *IRF1* (C)*, IFNB1* (D), and *CXCL10* (E) was quantified at 8 h post-infection. (F) HT1080 cells were mock-infected or infected with HSV-1 (MOI = 5), and WCLs were analyzed by immunoblotting at 8 h post-infection. (G–I) HT1080 cells were treated with H-151 (5 μM), and mock-infected or infected with HSV-1 (MOI = 5) for 8 h. The expression of *IRF1* (G)*, IFNB1* (H), and *CXCL10* (I) was quantified by RT-qPCR. (J) HT1080 cells were treated with H-151 (5 μM), and mock-infected or infected with HSV-1 (MOI = 5) for 8 h. WCLs were analyzed by immunoblotting. (K–L) HT1080 cells transduced with control sgRNA (Ctrl) or sgRNA targeting *MITA/STING.* The expression of *IRF1* was quantified by RT-qPCR (K), and WCLs were analyzed by immunoblotting (L).

HSV-1 is able to infect human HT1080 fibrosarcoma epithelial cells and elicit potent antiviral innate immune responses (He et al., 2021; Sharma et al., 2021; Song et al., 2020; Xie et al., 2023). We found that HSV-1 infection of HT1080 strongly induces IRF1 at both the transcriptional and protein levels (Fig. 1(A)–(B)), which is consistent with previous studies (Liu et al., 2024; Ru et al., 2014; Wang et al., 2020; Xie et al., 2018). The MITA/STING signaling pathway plays a critical role in the innate immune defense against HSV-1 (Hu et al., 2016; Ishikawa & Barber, 2008; Reinert et al., 2016; Sun et al., 2013; Wu et al., 2013; Zheng et al., 2022; Zhong et al., 2008). Therefore, we asked whether IRF1 induction by HSV-1 is dependent on MITA. Interestingly, CRISPR-mediated knockout of MITA significantly impaired the induction of IRF1 by HSV-1 (Fig. 1(C) and 1(F)). Consistent with previous reports (Chen et al., 2016; Hu & Shu, 2018), MITA knockout greatly blunted HSV-1-induced innate immune responses, as indicated by the reduced transcription of *IFNB1* and *CXCL10* (Fig. 1(D) –(E)). To further validate these results, we found that treatment with a specific MITA inhibitor H151 also greatly inhibited the induction of IRF1, as well as *IFNB1* and *CXCL10*, by HSV-1 (Fig. 1(G)–(J)). These data indicate that HSV-1 infection induces IRF1 and the induction of IRF1 by HSV-1 is partly dependent on MITA signaling. Next, we monitored the basal expression of IRF1 and found that knockout of MITA did not affect the basal level of IRF1 (Fig. 1(K)–(L)). Collectively, these data indicate that HSV-1 infection induces IRF1 and MITA contributes to the induction of IRF1 during HSV-1 infection.

### 1.2 MITA activation is sufficient to induce IRF1

**Fig. 2:**
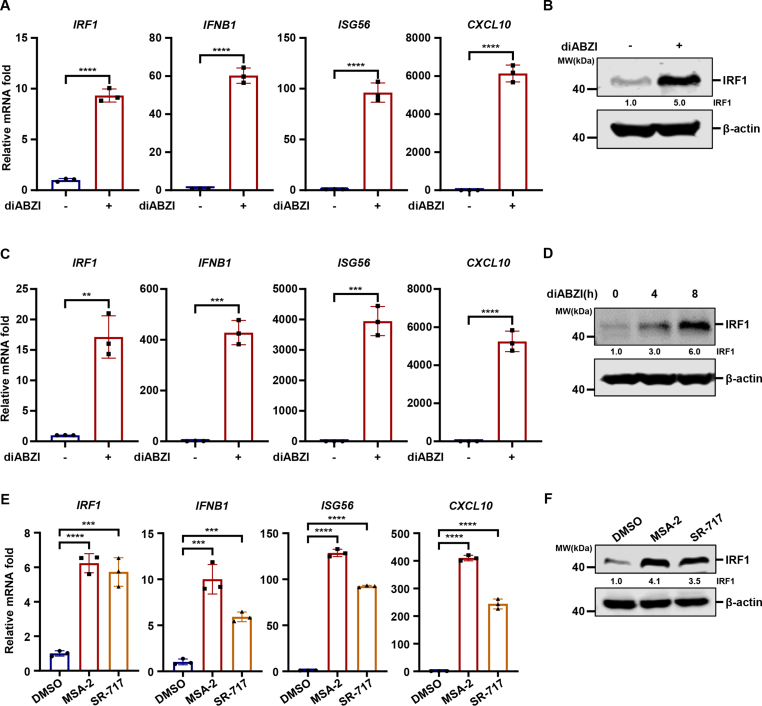
**MITA activation is sufficient to induce IRF1.** (A) HT1080 cells were stimulated with diABZI (2.5 μM), and the expression of *IRF1*, *IFNB1*, *ISG56,* and *CXCL10* was quantified by RT-qPCR at 6 h post-stimulation. (B) HT1080 cells were stimulated with diABZI (2.5 μM), and WCLs were analyzed by immunoblotting at 6 h post-stimulation. (C) THP-1 cells were stimulated with diABZI (2.5 μM), and the expression of *IRF1*, *IFNB1*, *ISG56,* and *CXCL10* was quantified by RT-qPCR at 6 h post-stimulation. (D) THP-1 cells were stimulated with diABZI (2.5 μM), and WCLs were analyzed by immunoblotting at the indicated time points post-stimulation. (E) HT1080 cells were stimulated with MSA-2 (20 μM) or SR-717 (10 μM), and the expression of *IRF1*, *IFNB1*, *ISG56,* and *CXCL10* was quantified by RT-qPCR at 8 h post-stimulation. (F) HT1080 cells were stimulated with MSA-2 (20 μM) or SR-717 (10 μM), and WCLs were analyzed by immunoblotting at 8 h post-stimulation.

Next, we investigated whether MITA activation is sufficient to induce IRF1. Treatment with a putative MITA agonist diABZI (Ramanjulu et al., 2018) significantly induced IRF1 transcription in HT1080 cells (Fig. 2(A)). Consistent with previous reports (Ramanjulu et al., 2018), diABZI activation of MITA potently activated antiviral innate immune responses, as indicated by the potent induction of *IFNB1*, *ISG56,* and *CXCL10* (Fig. 2(A)). Moreover, diABZI treatment greatly enhanced the protein level of IRF1 (Fig. 2(B)). THP-1 is a human monocyte cell line that is widely used in studies of innate immune regulation during HSV-1 infection (Christensen et al., 2016; Xia et al., 2019; Zhang et al., 2018). We confirmed that diABZI also induced IRF1 in THP-1 cells (Fig. 2(C)–(D)). Furthermore, treatment with two additional MITA agonists, MSA-2 and SR-717 (Chin et al., 2020; Pan et al., 2020), elicited strong innate immune responses and upregulated the expression of IRF1 (Fig. 2(E)–(F)). These data collectively indicate that MITA activation is sufficient to induce IRF1.

### 1.3 IRF1 restricts HSV-1 replication

**Fig. 3:**
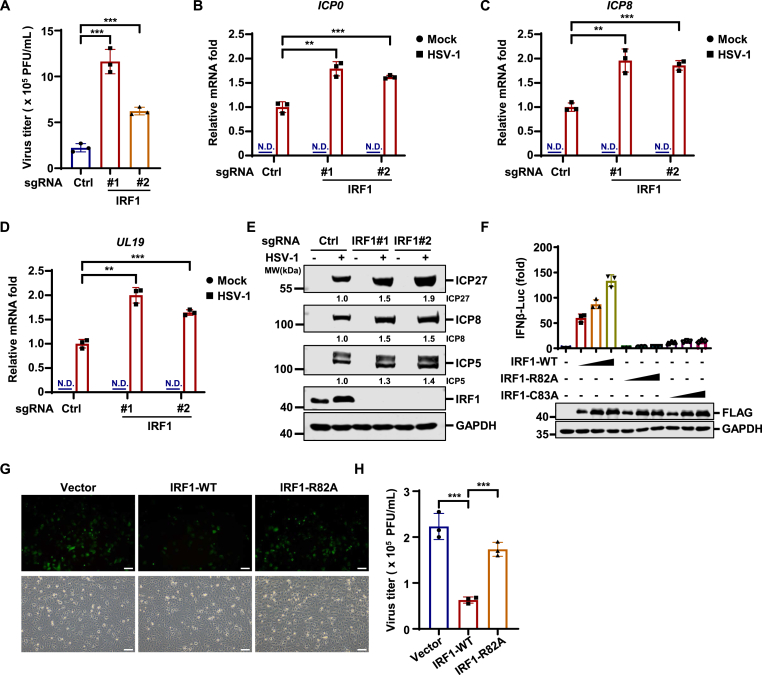
**IRF1 restrictsHSV-1replication.** (A) HT1080 cells transduced with control sgRNA (Ctrl) or sgRNA targeting *IRF1* were infected with HSV-1 (MOI = 0.01). Viral titers in the supernatants were quantified at 48 h post-infection. (B–E) THP-1 cells transduced with control sgRNA (Ctrl) or sgRNA targeting *IRF1* were mock-infected or infected with HSV-1 (MOI = 1). The expression of *ICP0, ICP8,* and *UL19* was quantified by RT-qPCR (B–D). The mock-infected samples were labeled as N.D. (not detected), and the signals from wild-type (WT) cells infected with HSV-1 were normalized to 1. WCLs were analyzed by immunoblotting at 8 h post-infection (E). (F) HEK293T cells were transfected with an IFN-β promoter reporter plasmid mixture with increasing amounts of *IRF1* expression plasmids (0, 0.1, 0.2, or 0.5 μg). Luciferase activities were measured at 24 h post-transfection. (G–H) HT1080 cells stably expressing vector control, IRF1-WT, or IRF1-R82A were infected with HSV-1-GFP (MOI = 0.05), and GFP expression was imaged at 24 h post-infection (G). Scale bars,100 μm. Viral titers in the supernatants were quantified at 24 h post-infection (H).

Next, we sought to investigate the role of IRF1 in HSV-1 replication. Our data indicate that HSV-1 viral titers in IRF1 knockout (KO) HT1080 cells were significantly higher than those in wild-type (WT) cells (Fig. 3(A)). Furthermore, viral gene transcription (*ICP0*, *ICP8*, and *UL19*) and viral protein expression (ICP27, ICP8, and ICP5) were increased in IRF1 KO cells (Fig. 3(B)–(E)). These data indicate that IRF1 deficiency promotes HSV-1 replication.

Since IRF1 is a transcription factor, we asked whether its DNA-binding activity is required for its antiviral activity against HSV-1. In order to identify a DNA-binding-deficient mutant of IRF1, we introduced mutations into the residues of IRF1 involved in DNA binding based on previous structural analysis (Escalante et al., 1998; Feng et al., 2021) and then monitored whether these mutants could activate IFNβ reporter activity. Wild-type IRF1 could potently activate IFNβ reporter activity. In contrast, the IRF-R82A mutant completely failed to activate IFNβ reporter, while the IRF-C83A mutant still weakly activated IFNβ reporter activity (Fig. 3(F)). These data suggest that IRF1-R82A disrupts the DNA-binding activity of IRF1, thereby blunting its transcriptional activity. IRF1 stable expression in HT1080 cells potently suppressed HSV-1-GFP replication, as demonstrated by reduced GFP fluorescence signaling and reduced viral titers in IRF1-expressing cells (Fig. 3(G)–(H)). In contrast, IRF1-R82A failed to restrict HSV-1 replication (Fig. 3(G)–(H)). Together, these data indicate that IRF1 suppresses HSV-1 replication dependent on its DNA-binding activity.

### 1.4 IRF1 amplifies HSV-1-triggered antiviral innate immunity

**Fig. 4:**
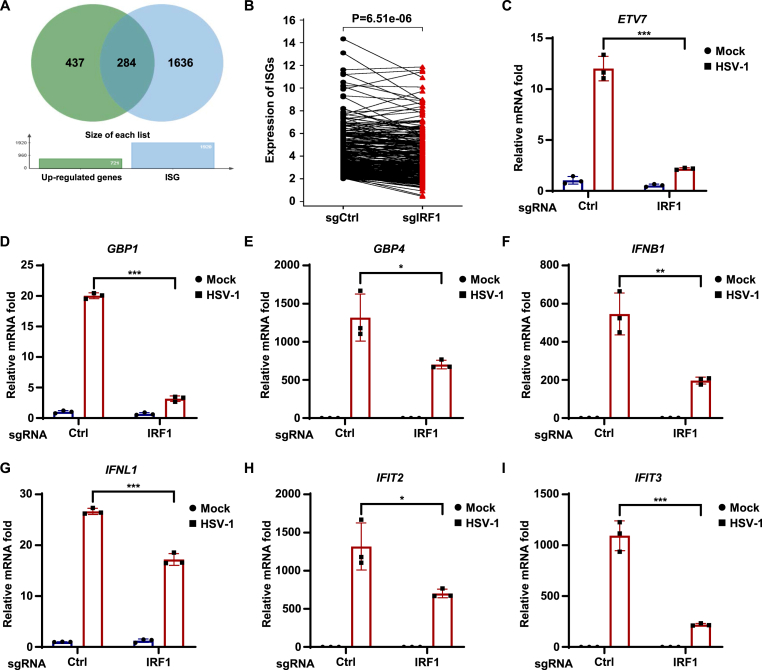
**IRF1 amplifiesHSV-1-triggeredantiviral innate immunity.** (A–B) THP-1 cells transduced with control sgRNA (Ctrl) or sgRNA targeting *IRF1* were mock-infected or infected with HSV-1 (MOI = 5) for 6 h. RNA-seq was performed, and a Venn diagram displayed significantly upregulated ISG genes after HSV-1 infection (A). The human ISG gene set was obtained from previous studies (OhAinle et al., 2018; Roesch et al., 2018). Paired line plots showed the expression levels of these upregulated ISGs in control and *IRF1* knockout cells (B). (C–I) THP-1 cells transduced with control sgRNA (Ctrl) or sgRNA targeting *IRF1* were mock-infected or infected with HSV-1 (MOI = 5) for 6 h. The expression levels of the indicated genes were quantified by RT-qPCR.

To investigate the role of IRF1 in HSV-1-triggered innate immune responses, we employed CRISPR to knockout *IRF1* in THP-1 cells and performed RNA sequencing with or without HSV-1 infection. Our analysis indicated that HSV-1 infection of WT THP1 cells increased the transcription of 721 genes, among which 284 genes were putative ISGs (Fig. 4(A)) (OhAinle et al., 2018; Roesch et al., 2018). These data are consistent with previous reports and demonstrate that HSV-1 infection induces strong antiviral innate immune responses (Zhang et al., 2018). Importantly, knockout of IRF1 greatly dampened the induction of the 284 ISGs when compared with WT THP1 cells (Fig. 4(B)), suggesting that IRF1 amplifies HSV-1-triggered antiviral innate immunity. Next, RT-qPCR quantification confirmed that the induction of the top differentially expressed ISGs (ETV7, GBP1, and GBP4) in the RNAseq was indeed significantly impaired in IRF1 KO cells (Fig. 4(C)–(E)). Moreover, we quantified the expression of type I IFNs (*IFNB1*), type III IFNs (*IFNL1*), and additional putative ISGs, and found that IRF1 KO also significantly impaired the induction of *IFNB1*, *IFNL1*, *IFIT2*, and *IFIT3* by HSV-1 (Fig. 4(F)–(I)). These data collectively indicate that IRF1 amplifies HSV-1-triggered antiviral innate immunity.

### 1.5 IRF1 interacts with IRF3 and promotes IRF3 recruitment to the promoters of ISGs as well as type I and III interferons

**Fig. 5:**
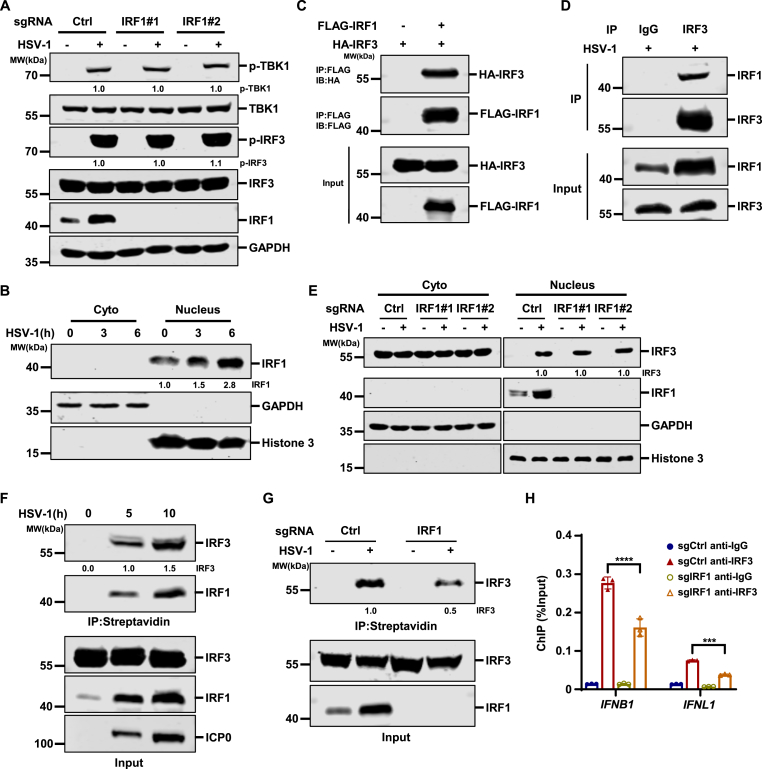
**IRF1 interacts with IRF3 and promotes IRF3 recruitment to ISG promoters.** (A) THP-1 cells transduced with control sgRNA (Ctrl) or sgRNA targeting *IRF1* were mock-infected or infected with HSV-1 (MOI = 5), and WCLs were analyzed by immunoblotting at 8 h post-infection. (B) THP-1 cells were mock-infected or infected with HSV-1 (MOI = 5), and nuclear and cytoplasmic fractions were isolated at the indicated time points and analyzed by immunoblotting. (C) HEK293T cells were transfected with the indicated plasmids, and WCLs were collected for immunoprecipitation with anti-FLAG affinity agarose. The input and precipitated samples were analyzed by immunoblotting. (D) HT1080 cells were infected with HSV-1 (MOI = 10) for 8 h. Co-immunoprecipitation was performed with the indicated antibodies, followed by immunoblotting analysis. (E) THP-1 cells transduced with control sgRNA (Ctrl) or sgRNA targeting *IRF1* were mock-infected or infected with HSV-1 (MOI = 10), and nuclear and cytoplasmic fractions were isolated at 8 h post-infection and analyzed by immunoblotting. (F) THP-1 cells were mock-infected or infected with HSV-1 (MOI = 10) for 5 or 10 h. Cell lysates were collected and pulldown assays were performed using a biotin-labeled ISG54 ISRE probe. The input and probe-bound proteins were analyzed with the indicated antibodies. (G) THP-1 cells transduced with control sgRNA (Ctrl) or sgRNA targeting *IRF1* were mock-infected or infected with HSV-1 (MOI = 10) for 8 h. Cell lysates were collected and pulldown assays were performed using a biotin-labeled ISG54 ISRE probe. The input and probe-bound proteins were analyzed using an anti-IRF3 polyclonal antibody, and the input samples were also analyzed using an anti-IRF1 monoclonal antibody. (H) THP-1 cells transduced with control sgRNA (Ctrl) or sgRNA targeting IRF1 were infected with HSV-1 (MOI = 10) for 10 h, followed by chromatin immunoprecipitation (ChIP) using an anti-IRF3 antibody or control IgG. IRF3 occupancy at the *IFNB1* and *IFNL1* promoter regions was assessed by qPCR.

Next, we examined the activation of antiviral signaling following HSV-1 infection in THP1 cells, and our results indicate that IRF1 KO did not affect the phosphorylation of TBK1 and IRF3 (Fig. 5(A)). IRF3 is known to be essential for HSV-1-induced innate immune activation (Stetson & Medzhitov, 2006; Zhong et al., 2008). Although IRF1 overexpression can activate IFNβ reporter activity, previous studies indicate that IRF1 plays an auxiliary role in innate immunity (Feng et al., 2021). In addition, cell fractionation analysis indicated that IRF1 largely localized in the nucleus both without and with HSV-1 infection (Fig. 5(B)). Therefore, we hypothesize that IRF1 may target IRF3 to regulate innate immunity.

Interestingly, IRF1 and IRF3 interacted with each other when overexpressed in HEK293T cells (Fig. 5(C)), and endogenous IRF1 formed a complex with IRF3 in HSV-1 infected THP1 cells (Fig. 5(D)). These data are consistent with previous studies (Wang et al., 2020) and suggest that IRF1 interacts with IRF3 to promote antiviral immunity. Moreover, IRF1 deficiency did not affect the nuclear translocation of IRF3 during HSV-1 infection (Fig. 5(E)). Notably, a biotinylated ISG54 promoter probe was able to pull down IRF3 only in HSV-1 infected cells (Meng et al., 2016; Nakaya et al., 2001; Yang et al., 2019). The binding of both IRF3 and IRF1 to the ISG54 promoter gradually increased during HSV-1 infection (Fig. 5(F)), while knockout of IRF1 significantly reduced IRF3 binding to the ISG54 promoter (Fig. 5(G)). Chromatin immunoprecipitation (ChIP) assays further confirmed that knockout of IRF1 markedly impaired the recruitment of IRF3 to the promoters of *IFNB1* and *IFNL1* (Fig. 5(H)). Together, these data suggest that IRF1 interacts with IRF3 and promotes its recruitment to the promoters of type I and type III interferons, as well as ISGs, thereby enhancing antiviral innate immunity.

**Fig. 6:**
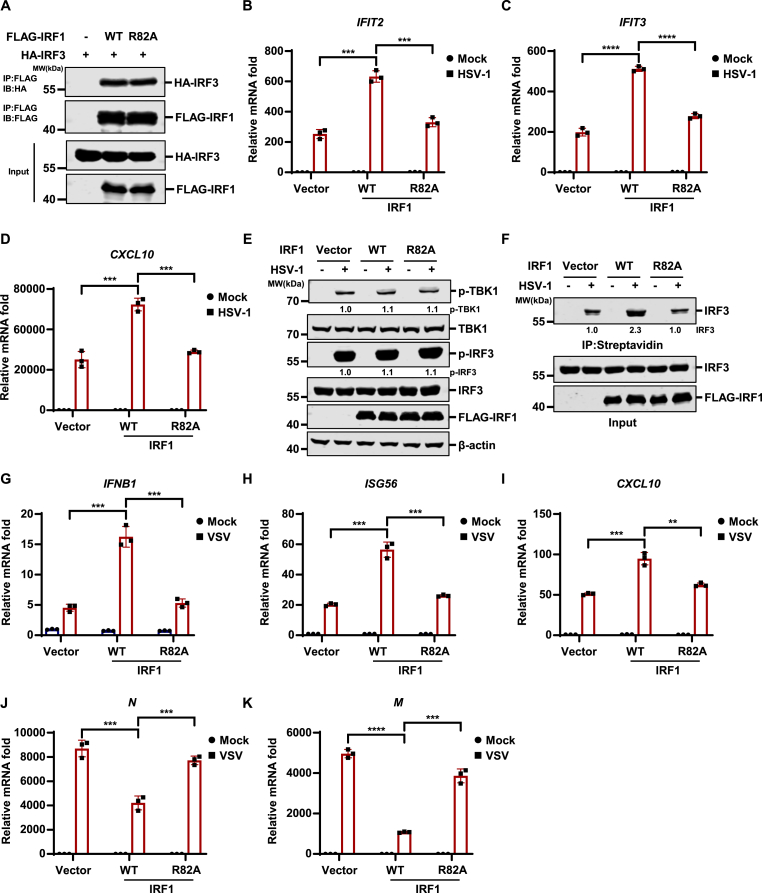
**IRF1 promotes antiviral innate immunity through itsDNA-bindingactivity.** (A) HEK293T cells were transfected with the indicated plasmids, and WCLs were collected for immunoprecipitation with anti-FLAG affinity agarose. The input and immunoprecipitated samples were analyzed by immunoblotting. (B–E) THP-1 cells stably expressing vector control, IRF1-WT, or IRF1-R82A were mock-infected or infected with HSV-1 (MOI = 5). The indicated genes were quantified by RT-qPCR (B–D), and WCLs were analyzed by immunoblotting at 8 h post-infection (E). (F) THP-1 cells stably expressing vector control, IRF1-WT, or IRF1-R82A were mock-infected or infected with HSV-1 (MOI = 10) for 8 h. Cell lysates were collected and pulldown assays were performed using a biotin-labeled ISG54 ISRE probe. The input and probe-bound proteins were analyzed by immunoblotting using an anti-IRF3 polyclonal antibody, and the input samples were also analyzed using an anti-IRF1 monoclonal antibody. (G–K) HT1080 cells stably expressing vector control, IRF1-WT, or IRF1-R82A were mock-infected or infected with VSV (MOI = 5) for 8 h. The expression levels of the indicated genes were quantified by RT-qPCR.

The DNA-binding deficient IRF1-R82A mutant also interacted with IRF3 (Fig. 6(A)). While WT IRF1 significantly promoteed HSV-1-induced innate immune responses, as indicated by the enhanced transcription of *IFIT2*, *IFIT3*, and *CXCL10* in IRF1-expressing cells compared with control cells (Fig. 6B–D), the induction of these innate immune genes by IRF1-R82A was substantially impaired compared with WT IRF1 (Fig. 6(B)–(D)). Moreover, IRF1-R82A expression also did not impact the phosphorylation of TBK1 and IRF3 induced by HSV-1 (Fig. 6(E)). Notably, IRF1-R82A expression failed to promote the binding of IRF3 to the ISG54 promoter as WT IRF1 did (Fig. 6(F)). These results suggest that the DNA-binding activity of IRF1 is required to facilitate the recruitment of IRF3 to the ISG promoters during HSV-1 infection.

Given that IRF1 interacts with IRF3 and promotes its DNA-binding activity, we hypothesize that IRF1 may also function as an amplifier of innate immune defenses against RNA viruses. Indeed, stable expression of WT IRF1, but not the DNA-binding deficient R82A mutant, enhanced VSV-induced innate immune responses (Fig. 6(G)). Moreover, stable expression of WT IRF1, but not the R82A mutant, restricted VSV replication (Fig. 6(H)). These data suggest that IRF1 also amplifies RNA virus-induced innate immunity to restrict viral replication.

Collectively, these data indicate that IRF1 interacts with IRF3 and promotes IRF3 recruitment to the promoters of ISGs to promote antiviral innate immunity.

## 2 Discussion

In this study, we have uncovered a pivotal role for IRF1 in amplifying HSV-1-induced antiviral innate immune responses through a feed-forward mechanism. Our findings indicate that the MITA/STING signaling pathway contributes to IRF1 induction upon HSV-1 infection and IRF1 enhances antiviral innate immunity by interacting with IRF3, promoting its recruitment to the promoters of ISGs. The interaction between IRF1 and IRF3 is critical for the optimal activation of innate immune responses that restrict viral replication. Consistently, we have found that the DNA-binding activity of IRF1 is critical for promoting HSV-1-induced innate immune responses and restricting HSV-1 replication.

Previous studies have shown that IRF1 is induced by a variety of stimuli, including viral infections, with broadly antiviral roles (Rahlf & Tarakanova, 2025; Wang et al., 2020). Furthermore, IRF1 plays a critical role in regulating the acute infection of murine gammaherpesvirus 68 (MHV68) and the establishment of chronic infection (Jondle et al., 2020, 2021, 2022; Mboko et al., 2017). In addition, IRF-1 restricts HCV replication through modulating interferon-stimulated gene-mediated antiviral responses (Kanazawa et al., 2004). Interestingly, IFNγ-primed IRF1 expression more efficiently restricts VACV replication in glucose-rich culture conditions that favors aerobic glycolysis (Chang et al., 2024). Notably, a recent study reports that IRF1 restricts HSV-1 replication in astrocytes (Liu et al., 2024). IRF1 has also been reported to promote the phosphorylation of IRF3 by disrupting the interaction between IRF3 and PP2A, a major phosphatase that dephosphorylates and inactivates phosphorylated IRF3 (Long et al., 2014), thereby promoting antiviral innate immunity and inhibiting the replication of VSV and NDV (Wang et al., 2020). Interestingly, knockout of MITA or blockade of MITA signaling failed to completely abolish the induction of IRF1 by HSV-1, which is consistent with previous reports that HSV-1 infection can activate RIG-I signaling (Chiang et al., 2018; Zhao et al., 2016), which may also contribute to IRF1 induction.

While the cytoplasmic role of IRF1 in disrupting the PP2A-IRF3 interaction has been proposed, our data reveal that IRF1 is predominantly localized in the nucleus. Integrating these findings, we propose a dual mechanism whereby IRF1 promotes IRF3 phosphorylation in the cytoplasm by blocking PP2A, while in the nucleus, IRF1 binds to IRF3 and enhances its recruitment to ISG promoters, amplifying antiviral immune responses during HSV-1 infection. It is worth mentioning that since the DNA-binding activity of IRF1 is essential for its transcriptional regulation, it is challenging to definitively separate the effects of the DNA-binding activity of IRF1 from the potential contributions of downstream IRF1-induced genes in innate immune regulation. The potential involvement of IRF1 downstream genes in antiviral innate immunity during HSV-1 infection warrants further investigation.

In addition to its induction by viral infections, basal expression of IRF1 has been shown to drive the optimal expression of a battery of antiviral effector genes that defend against various pathogenic RNA viruses (Panda et al., 2019; Yamane et al., 2019). Basal expression of IRF1 sets up an antiviral state in cells, preparing them to respond rapidly to infections (Panda et al., 2019; Yamane et al., 2019). This basal IRF1 expression could also have a role in controlling HSV-1 infection during the early stages of infection. The relative contributions of basal versus induced IRF1 in HSV-1 infection require further investigation.

In summary, our study defines IRF1 as an amplifier in HSV-1-induced antiviral innate immunity. MITA signaling contributes to the induction of IRF1 during HSV-1 infection, and IRF1 restricts HSV-1 replication dependent on its DNA-binding activity. By interacting with IRF3, IRF1 enhances IRF3 recruitment to ISG promoters and boosts antiviral gene expression. These findings provide valuable insights into the complex interplay between viral pathogens and the host innate immune system, highlighting the potential for modulating IRF1 in antiviral interventions.

## 3 Materials and methods

### 3.1 Cell culture and viruses

HEK293T (ATCC-CRL-3216), HT1080 (ATCC-CCL-121), and VERO (ATCC-CCL-81) cells were cultured in Dulbecco’s Modified Eagle’s Medium (DMEM; Gibco) supplemented with 10% fetal bovine serum (FBS; LONSERA, Shanghai, China) and 1% penicillin-streptomycin (Gibco). THP-1 (ATCC) cells were cultured in RPMI-1640 (Gibco) supplemented with 10% FBS and 1% penicillin–streptomycin.

Herpes simplex virus type 1 (HSV-1) (F strain) (Xie et al., 2023), HSV-1-GFP (F strain), and VSV (Sun et al., 2020) were propagated using VERO cells. Virus titers were determined by standard plaque assays using VERO cells.

### 3.2 Constructs

For transient expression in mammalian cells, *IRF1* and its mutants (R82A and C83A) were constructed into pEF-EF1α-FLAG-N vector. *IRF3* was constructed into the pEF-EF1α-HA-N vector. For stable cell line generation, *IRF1* and its mutant R82A were subcloned into pCDH-CMV-EF1α-Puro (Takara) with a FLAG tag. The plasmids pRL-TK-Renilla and pGL3-IFN-β-Luciferase were kindly provided by Dr. Pinghui Feng (University of Southern California) (Minassian et al., 2015). sgRNAs targeting *IRF1* and *MITA/STING* (Zhang et al., 2023) were constructed into Lenti-CRISPRv2 (52961, Addgene). The following sgRNA sequences were used in this study:

sgIRF1#1: 5’-CACCGCTCAGCTGTGCGAGTGTAC-3’

sgIRF1#2: 5’-CACCGCATGGCTGGGACATCAACA-3’

sg MITA/STING: 5’-CCTGCCTGGTGACCCTTTGGGGG-3’

### 3.3 Generation of stable cell lines

Lentivirus was produced by transient transfection of HEK293T cells using a second-generation packaging system (Zhang et al., 2015). Briefly, HEK293T cells were seeded in 10 cm dishes and transfected with the lentiviral expression plasmids, along with the packaging plasmids psPAX2 and pVSVG. The culture medium was replaced with fresh complete DMEM after 6 h. Forty-eight hours post-transfection, lentivirus-containing supernatants were collected, filtered through a 0.45-μm filter, and stored at −80 °C.

For the generation of stable cell lines (Wang et al., 2024b, 2025), target cells were seeded in 6-well plates and infected with lentiviral supernatants supplemented with 8 μg/mL polybrene (Sigma-Aldrich). After 6 h, the infection medium was replaced with fresh complete growth medium. Infected cells were selected with puromycin (1 μg/mL) for 3–5 days, and the stable cells were maintained in complete growth medium with puromycin (1 μg/mL).

## 4 Antibodies and other regents

The following antibodies and reagents were used for immunoblotting and immunoprecipitation: Mouse anti-FLAG monoclonal antibody (1:10,000, Dia-An Biotechnology, catalog no. 2064); Mouse anti-HA monoclonal antibody (1:5000, Dia-An Biotechnology, catalog no. 2063); Mouse anti-β-actin monoclonal antibody (1:5000, Dia-An Biotechnology, catalog no. 2060); Mouse anti-GAPDH monoclonal antibody (1:1000, Santa Cruz, sc-47724); Histone H3 antibody (1:1000, Santa Cruz, sc-517576); Rabbit anti-MITA/STING polyclonal antibody (1:5000, Proteintech, catalog no. 19851-1-AP); Rabbit anti-IRF3 polyclonal antibody (1:1000, Proteintech, catalog no. 11312-1-AP); Rabbit anti-TBK1 monoclonal antibody (1:1000, Cell Signaling Technology, catalog no. 3504); Rabbit anti-phospho-IRF3 (S386) monoclonal antibody (1:1000, Abcam, AB76493); Rabbit anti-phospho-TBK1 (S172) monoclonal antibody (1:1000, Cell Signaling Technology, catalog no. 5483); Rabbit anti-IRF1 monoclonal antibody (1:1000, Cell Signaling Technology, catalog no. 8478); Rabbit IgG (Proteintech, catalog no. 20010049); Mouse anti-ICP0 monoclonal antibody (1:1000, Santa Cruz, sc-53070); Mouse anti-ICP8 monoclonal antibody (1:1000, Santa Cruz, sc-53329); Mouse anti-ICP27 monoclonal antibody (1:1000, Santa Cruz, sc-69806); Mouse anti-ICP5 monoclonal antibody (1:1000, Santa Cruz, sc-56989); IRDye 800CW Goat anti-Rabbit and Goat anti-Mouse secondary antibodies (1:10,000, LI-COR); Anti-FLAG beads (Dia-An Biotechnology); Protein A/G agarose (GE healthcare).

The chemical reagents used in this study include puromycin (InvivoGen), diABZI (Selleck), H-151 (MedChemExpress), MSA-2 (MedChemExpress) and SR-717 (MedChemExpress).

### 4.1 Reporter assay

Luciferase reporter assays were performed using a dual luciferase reporter assay system (Promega) (Wang, Tian, et al., 2024). HEK293T cells in 24-well plates were transfected with a plasmid mixture containing 50 ng of the plasmid expressing IFN-β firefly luciferase reporter, 20 ng of the plasmid expressing TK-Renilla luciferase reporter, and different amounts of IRF1 expression plasmid (WT or mutants). At 24 h post-transfection, cells were harvested and cell lysates were prepared. Luciferase activities were measured according to the manufacturer’s instructions (Promega).

### 4.2 RNA extraction and RT-qPCR

Human THP-1 or HT1080 cells were either infected with HSV-1 (MOI = 1 or 5), VSV (MOI = 5) or stimulated with diABZI (2.5 μM), MSA-2 (20 μM) or SR-717 (10 μM). At the indicated time points, cells were washed with ice-cold phosphate-buffered saline (PBS), and total RNA was extracted using TRIzol reagent (Takara) (Tian et al., 2023). The extracted RNA was treated with DNase I (New England Biolabs) to eliminate genomic DNA contamination. Reverse transcription was performed using 1 μg of total RNA with HiScript II Reverse Transcriptase (Vazyme, Nanjing, China) according to the manufacturer’s instructions. qPCR was conducted using the SYBR Green qPCR Master Mix (Bimake, Shanghai, China) on a Bio-Rad CFX Connect Real-Time PCR Detection System. Relative expression levels of target genes were normalized to the housekeeping gene *ACTB*. The primers used for qPCR are listed in Supplementary File 1.

### 4.3 RNA-seq

THP-1 cells transduced with control sgRNA (Ctrl) or sgRNA targeting *IRF1* were mock-infected or infected with HSV-1 (MOI = 5) for 6 h (two biological replicates per sample). Total RNA was extracted using the TRIzol reagent (Takara) according to the manufacturer's instructions. Subsequently, mRNA was enriched using oligo (dT) magnetic beads, and sequencing libraries were constructed using an Illumina platform library preparation kit. Paired-end sequencing was performed on the Illumina NovaSeq high-throughput sequencing platform (Annoroad Gene Technology, Beijing, China). The clean reads were aligned to the reference genome using HISAT2 (v.2.2.1), followed by transcript quantification with featureCounts (v.2.0.3). Gene expression levels were calculated as FPKM/TPM, and differential expression analysis was conducted using DESeq2 (v.1.36.0) with default settings. Differentially expressed genes (DEGs) were defined as those with log_2_FoldChange ≥2 and *p*-value ≤0.05.

### 4.4 Immunoprecipitation

To evaluate the interaction between IRF3 and IRF1 WT/R82A, HEK293T cells were transfected with the specified expression plasmids and collected 24 h post-transfection. Cells were lysed in NP-40 lysis buffer (150 mM NaCl, 50 mM Tris-HCl, pH 7.4, 1% NP-40, 1 mM EDTA) inhibitors supplemented with a protease inhibitor cocktail, and the lysates were centrifuged at 14,000 g for 10 min at 4 °C to collect the supernatant. The supernatant was incubated with 10 μL of anti-FLAG beads (Dia-An Biotechnology) at 4 °C for 4 h. For endogenous protein immunoprecipitation, HT1080 cells were infected with HSV-1 (MOI = 5) and harvested 8 h post-infection. Whole-cell lysates were prepared as described previously (Zhou et al., 2024). The lysates were incubated overnight with either 2 μg of rabbit IgG (Proteintech) or rabbit ani-IRF3 polyclonal antibody (Proteintech), followed by the addition of 10 μL Protein A/G agarose (GE Healthcare) and further incubation on a rotator at 4 °C for 4 h. The beads were then extensively washed with NP-40 lysis buffer, and bound proteins were eluted by boiling in SDS sample buffer at 95 °C for 10 min. The samples were separated by SDS-PAGE and analyzed by immunoblotting.

### 4.5 Cell fractionation

THP-1 cells were infected with HSV-1 (MOI = 1 or 5) and harvested 6 h post-infection. Cells were lysed on ice for 10 min using sucrose lysis buffer (10 mM HEPES, pH 7.9, 340 mM sucrose, 3 mM CaCl_2_, 2 mM magnesium acetate, 0.1 mM EDTA, 0.5% NP-40, supplemented with a protease inhibitor cocktail). The samples were then centrifuged at 3500 g for 5 min at 4 °C, and the supernatant was collected as the cytoplasmic fraction. The remaining pellets were washed twice with sucrose buffer without NP-40. After washing, 1 × SDS loading buffer was added to the pellets, and the samples were boiled at 95 °C for 20 min. Proteins were subsequently separated by SDS-PAGE and analyzed by Western blotting.

### 4.6 Biotin DNA pull-down

THP-1 cells were infected with HSV-1 (MOI = 5) for 5, 8 or 10 h, then harvested and washed once with PBS. The cells were lysed on ice for 15 min using lysis buffer (20 mM HEPES, pH 7.9, 150 mM NaCl, 1 mM EDTA, 1 mM DTT, 1% NP-40, supplemented with protease inhibitors). After centrifugation, the supernatant containing total protein was collected. Streptavidin agarose beads were pre-incubated with a biotin-labeled triple-repeat ISG54 ISRE probe (5’-GGGAAAGTGAAACTAGGGAAAGTGAAACTAGGGAAAGTGAAACTA-3’) (Meng et al., 2016; Nakaya et al., 2001; Yang et al., 2019) and then incubated with the cell lysate at 4 °C for 4 h to enrich DNA-binding proteins. After washing extensively with lysis buffer to remove non-specific interactions, the agarose beads were resuspended in 1 × SDS loading buffer and boiled at 95 °C for 20 min. The released proteins were separated by SDS-PAGE and analyzed by immunoblotting.

### 4.7 Chromatin immunoprecipitation (ChIP)

THP-1 cells transduced with control sgRNA (Ctrl) or sgRNA targeting *IRF1* were infected with HSV-1 (MOI = 10) for 10 h. Chromatin immunoprecipitation (ChIP) assays were performed using the Beyotime ChIP Kit (P2083S) according to the manufacturer’s instructions. Briefly, cells were cross-linked with 1% formaldehyde at 37 °C for 10 min, and the reaction was quenched with 0.125 M glycine for 5 min. The cross-linked DNA-protein complexes were digested with micrococcal nuclease (MNase) to generate DNA fragments ranging from 200 to 1000 bp. Cell lysates were then immunoprecipitated with 2 μg of IRF3 antibody (Proteintech, 11312-1-AP) or rabbit IgG (Proteintech, 30000-0-AP) as a control. The immunoprecipitated DNA-protein complexes were processed to reverse cross-linking. The samples were then digested with 30 μL of proteinase K (20 mg/mL, Solarbio) and 30 μL of RNase A (10 mg/mL, Solarbio), and DNA was purified by phenol-chloroform extraction (Tian et al., 2023). ChIP DNA levels were normalized to input DNA. In each experiment, and all samples were analyzed in triplicate. The primers used for ChIP-qPCR are listed in Supplementary File 1.

### 4.8 Statistical analysis

All experiments were performed at least three times independently, and data are presented as mean ± standard deviation (mean ± SD). Statistical analysis was conducted using GraphPad Prism (version 8.0). Differences between groups were assessed using a two-tailed Student’s *t*-test for pairwise comparisons or one-way/multi-way analysis of variance (ANOVA) for multiple group comparisons. Statistical significance is indicated as follows: ∗*p* < 0.05, ∗∗*p* < 0.01, ∗∗∗*p* < 0.001, ∗∗∗∗*p* < 0.0001, and N.S. indicates no statistical significance.

## CRediT authorship contribution statement

**Ming Gao:** Writing – original draft, Methodology, Investigation, Formal analysis, Conceptualization. **Yining Qi:** Investigation, Formal analysis, Conceptualization. **Junjie Zhang:** Writing – original draft, Supervision, Funding acquisition, Conceptualization.

## Declaration of competing interest

The authors declare that they have no known competing financial interests or personal relationships that could have appeared to influence the work reported in this paper.
