# Supplementary material for: IRF1 amplifies HSV-1-triggered antiviral innate immunity in a feed-forward manner
Source: Cell Insight. 2025 May 22;4(4):100255. doi: 10.1016/j.cellin.2025.100255 (PMC12205802; doi:10.1016/j.cellin.2025.100255)
Supplement: Multimedia component 1 [file mmc1.pdf]

Q-PCR primers for human genes

gene	forward（5’-3’）	reverse（5’-3’）
ACTB	GTTGTCGACGACGAGCG	GCACAGAGCCTCGCCTT
IFNB1	CAGGAGAGCAATTTGGAGGA	CTTTCGAAGCCTTTGCTCTG
ISG56	TCTCAGAGGAGCCTGGCTAA	TGACATCTCAATTGCTCCAG
CXCL10	CACCATGAATCAAACTGCGA	GCTGATGCAGGTACAGCGT
IFIT2	AAGCACCTCAAAGGGCAAAAC	TCGGCCCATGTGATAGTAGAC
IFIT3	TCAGAAGTCTAGTCACTTGGGG	ACACCTTCGCCCTTTCATTTC
IRF1	ACCCTGGCTAGAGATGCAGA	TGCTTTGTATCGGCCTGTGT
IFNL1	AACTGGGAAGGGCTGCCACATT	GGAAGACAGGAGAGCTGCAACT
ETV7	CAAGATCTTCCGAGTTGTGGA	GTTCACCCGGTTCTTGTGAT
GBP1	TATTGCCCACTATGAACAGCAGAT	TAGCTGGGCCGCTAACTCC
GBP4	TAAGCGGCTTTCAGAGCACC	GACCTCGTTTGCCTTAACTCC

Q-PCR primers for viral genes

gene	forward（5’-3’）	reverse（5’-3’）
ICP0	GTCGCCTTACGTGAACAAGAC	GTCGCCATGTTTCCCGTCTG
ICP8	TGGCTTTTCGGACTACACCC	TTCGAAGGCCGTGAACGTAA
UL19	GGACCGCTTTGTGACTGAGA	TGAGCGTGAAGTTTACCCCC
N	GATAGTACCGGAGGATTGACGACTA	AACCATCCGAGCCATTCGA
M	TGGAGTTGACGAGATGGACAC	TTTCCCTGCCATTCCGATGT

ChIP-qPCR primers

gene	forward（5’-3’）	reverse（5’-3’）
IFNB1	AGAAACTACTAAAATGTAA	TGGGTATGGCCTATTTATAT
IFNL1	CTTCCTCTCTGCCACTCAGG	ACTGCTTCCCCAGCGGCATG
